# Constructing Heterogeneous Photocatalysts Based on Carbon Nitride Nanosheets and Graphene Quantum Dots for Highly Efficient Photocatalytic Hydrogen Generation

**DOI:** 10.3390/ma15155390

**Published:** 2022-08-05

**Authors:** Yong Wang, Chengxin Zeng, Yichen Liu, Dingyi Yang, Yu Zhang, Zewei Ren, Qikun Li, Jian Hao, Wen Hu, Yizhang Wu, Rusen Yang

**Affiliations:** 1 Academy of Advanced Interdisciplinary Research, School of Advanced Materials and Nanotechnology, Xidian University, Xi’an 710126, China; 2 Department of Physics, Shaanxi University of Science and Technology, Xi’an 710021, China; 3 State Key Laboratory of High-Efficiency Utilization of Coal and Green Chemical Engineering, Ningxia University, Yinchuan 750021, China; 4 National Laboratory of Solid State Microstructures, Collaborative Innovation Center of Advanced Microstructures and Jiangsu Provincial Key Laboratory for Nanotechnology, Nanjing University, Nanjing 210093, China

**Keywords:** heterojunctions, photogenerated carriers, hydrogen generation

## Abstract

Although graphitic carbon nitride nanosheets (CNs) with atomic thickness are considered as promising materials for hydrogen production, the wide band gap (3.06 eV) and rapid recombination of the photogenerated electron–hole pairs impede their applications. To address the above challenges, we synergized atomically thin CNs and graphene quantum dots (GQDs), which were fabricated as 2D/0D Van der Waals heterojunctions, for H_2_ generation in this study. The experimental characterizations indicated that the addition of GQDs to the π-conjugated system of CNs can expand the visible light absorption band. Additionally, the surface photovoltage spectroscopy (SPV) confirmed that introducing GQDs into CNs can facilitate the transport of photoinduced carriers in the melon chain, thus suppressing the recombination of charge carriers in body. As a result, the H_2_ production activity of the Van der Waals heterojunctions was 9.62 times higher than CNs. This study provides an effective strategy for designing metal-free Van der Waals hetero-structured photocatalysts with high photocatalytic activity.

## 1. Introduction

Since the TiO_2_ discovery as a photocatalyst for water splitting, different semiconductor materials with specific energy-band positions have been employed as photocatalytic materials for pollutant degradation, H_2_ production from water splitting, and carbon dioxide reduction [1,2,3,4,5,6,7,8,9]. Photocatalysts are considered to be an efficient technique to generate hydrogen energy, while also addressing environmental and energy issues. [4,5,10,11,12]. However, many of the semiconductor materials have a limited visible light-absorption range, due to the large band gaps caused by the usage of sunlight [13,14,15,16,17]. Additionally, the poor separation efficiency and rapid recombination of the photoinduced electron–hole pairs are other important factors limiting photocatalytic hydrogen production [18,19,20,21].

Two-dimensional semiconductor materials have a larger specific surface area, more reactive sites on the surface, and shorter photogenerated carrier-separation pathways than bulk materials [22,23,24,25]. As a result of their exceptional features, the 2D semiconductor materials have considerable application potential in the area of photocatalytic water splitting [26,27,28,29,30,31,32]. As a non-metallic photocatalyst, the atomically thin graphitic carbon-nitride nanosheets (CNs) are considered to be promising emerging photocatalysts because of their simple synthesis, high stability, and nontoxicity [6,24,27,28]. Although their conduction band and valence band position meet the requirements for overall water splitting, they can only respond to light of a wavelength below 419 nm owing to the large band gap of 3.06 eV, and therefore miss the most visible light [6,27,33,34,35]. Additionally, low photogenerated carrier separation efficiency and the quick recombination of photoinduced electron–hole pairs are still the key obstacles restricting the application of CNs.

To address these challenges, a composite of semiconductor photocatalytic materials and graphene systems can be used to broaden the absorption range of visible light and enhance the light absorption intensity of its materials [35,36,37,38,39,40,41,42,43]. Due to graphene’s metallic properties, the heterojunctions constructed are transformed into Schottky heterojunctions. However, as Schottky heterojunctions, the carriers are difficult to separate due to the large energy barrier at the interface, which further limits their practical application [44,45]. Therefore, determining the graphene materials that possess semiconductor properties is necessary.

Fortunately, graphene quantum dots (GQDs) as 0-dimensional nanomaterials have some special advantages, such as high dispersibility, a wide variety of active sites (edges, functional groups, dopants, etc.), and better tunability of the chemical and physical properties [46,47,48]. Therefore, the GQDs have garnered considerable interest in the sectors of the conversion and storage of energy, electrical/optical/chemical catalysis, among others. More importantly, GQDs have semiconductor properties due to their quantum confinement effect. Therefore, we constructed heterogeneous photocatalysts based on CNs and GQDs. The experimental characterization shows that the absorption edge of the sample is red-shifted, due to the introduction of the GQDs. When Pt is employed as a cocatalyst, the hydrogen production performance of the CNs/GQDs-3 reaches 4.99 mmol g^−1^ h^−1^, which is 9.62 times greater than that of the CNs. The creation of a CN heterojunction increases the usage of visible light and facilitates the separation of the photogenerated carriers, consequently considerably boosting the hydrogen production capacity of the catalyst.

## 2. Synthesis and Characterization

### 2.1. Preparation of CNs/GQDs Samples

Preparation of the CNs: First, 10 g of urea was put into a crucible that was covered with another crucible, then the crucibles were wrapped with tin foil and placed in a box furnace. At a heating rate of 0.5 °C min^−1^, the furnace temperature was raised to 550 °C in air, held for three hours; the bulk CN was obtained after cooling. Secondly, the bulk CN was ground into powders with a mortar, then 100 mg of the powder was placed in a large crucible and flattened. The crucible was put into a box furnace. At a heating rate of 5 °C min^−1^, the furnace temperature was raised to 520 °C in air, held for two hours, and the sample was then cooled to room temperature in the oven, and the graphitic carbon nitride nanosheets were obtained (CNs).

Preparation of the GQDs: A total of 2 g of the dried carbon block was weighed and then put into a reactor that containing 300 mL of 15 M concentrated nitric acid. Next, the solution from the previous step was maintained at 140 °C for 24 h for the reaction to occur and then raised to 180 °C to produce the dry powders. The product was dissolved in 1 L of distilled water and then the dispersion was centrifuged at 10,000 rpm for 30 min to obtain a supernatant. The large particles were filtered out of the supernatant, and the resulting dispersion was then freeze-dried to produce the gray GQDs powder.

Preparation of the CNs/GQDs samples: A total of 200 mL of distilled water was measured into a beaker and the pH was adjusted to four with hydrochloric acid. A total of 200 mg of CNs was added and stirred for 1 h to positively charge the surface of the CNs. The GQDs dispersion at a concentration of 1 mg/mL was added and mixed for 24 h to establish electrostatic adsorption between the GQDs and CNs. Then, the dispersion was centrifuged at 9000 rpm and repeated several times until the solution was neutral. The solution was then freeze-dried and the product was annealed in an argon atmosphere at 300 °C for 1 h. The samples were named CNs/GQDs-X (X = 1, 3, 5, X depending on the mass ratio of the GQDs to the CNs).

### 2.2. Characterization

The TEM was recorded on a JEM-2100F microscope (JEOL, Tokyo, Japan). The AFM and SKPM were measured by an atomic force microscope (OXFORD Cypher). The XRD data were measured by a D8+ Advance X-ray diffractometer (Bruker, Munich, Germany). The FTIR spectra were measured by a Nicolet+iS+50 Fourier Infrared spectrometer. The XPS measurements were recorded by a Thermo Scientific K-Alpha+ (ThermoFisher, Waltham, MA, USA). The UV-vis absorption data were performed on a Perkin Elmer Lambda UV-vis spectrophotometer. The PL spectra were measured by a 1J1-0015 spectrophotometer.

### 2.3. Electrochemical Measurements

The preparation of the working electrodes: A total of 5 mg of the CNs and CNs/GQDs-3 were dispersed in 5ml Alpha-terpineol, respectively. The dispersion was then sonicated for two hours to ensure a uniform slurry. Using a spatula to distribute the slurry uniformly over the 2.5 × 2.5 cm^2^ ITO glass electrodes. The electrodes were vacuum dried at 80 °C for 8 h to obtain the working electrodes. The tests were performed in a three-electrode system, utilizing a platinum sheet as the counter electrode, and using Ag/AgCl electrode as the reference electrode.

### 2.4. Photocatalytic Activity Measurement

A total of 25 mg of the sample was weighed and then distributed in 100 mL of deionized water which contained 10% TEOA. The H_2_PtCl_6_ with a mass fraction of 3% was added to the above dispersion as a co-catalyst. The dispersion was illuminated for 4 h with a 300 W xenon lamp (λ > 420 nm), and a data point was recorded every hour.

The apparent quantum efficiency (AQE) was measured using three LED lamps (420 nm, 550 nm, and 600 nm) and an optical power meter (Thorlabs), and was calculated based on the following equation:

$$\mathrm{AQE}=\frac{N_{e}}{N_{q}}\times 100\%=\frac{2\times M\times N_{A}\times h\times c}{S\times P\times t\times \lambda }$$

where *N_e_* is the amount of reaction electrons; *N_p_* is the incident photons; *M* is the amount of H_2_ molecule; *N_A_* is Avogadro constant; *h* is the Planck constant; *c* is the speed of light; *S* is the irradiation area; *P* is the intensity of the irradiation; *t* is the photoreaction time; and *λ* is the wavelength of the monochromatic light.

## 3. Results

### 3.1. Morphological Characterization

**Figure 1:**
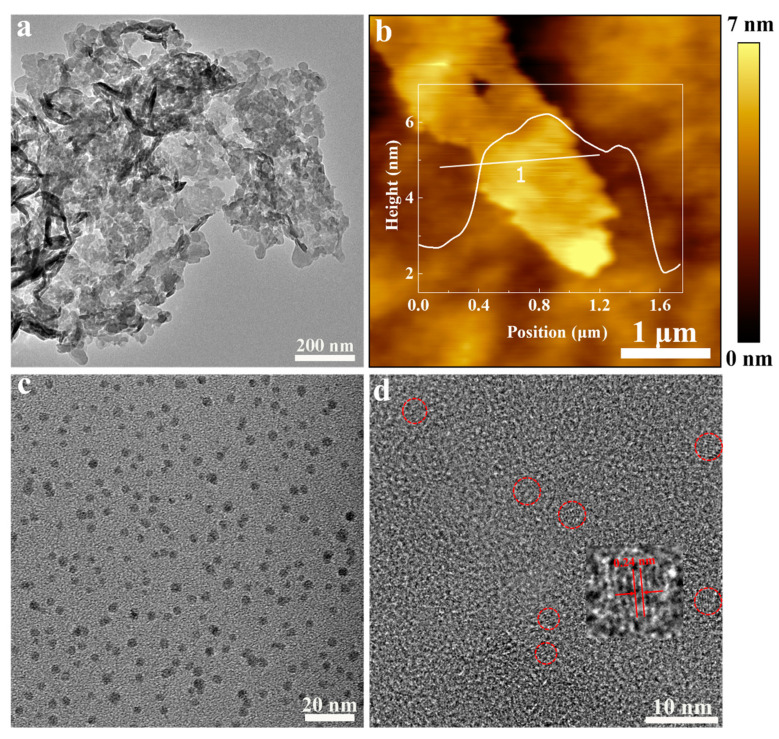
(**a**) TEM image of CNs; (**b**) The atomic force microscope image of CNs, and the white inset is the corresponding height profile along the white line; (**c**) TEM image of GQDs; and (**d**) High resolution transmission electron microscope (HRTEM) image of CNs/GQDs-3. The red circle and the inset are the location and magnification of GQDs, respectively.

The CNs were obtained by the deamination condensation of urea. The TEM result in Figure 1a shows that the CNs are flake-like and have a lateral dimension of about 1 μm. The thickness of the CNs ranges from 3 nm to 3.5 nm (Figure 1b). The graphene quantum dots (GQDs) were prepared by the oxidation-cutting method [46]. Figure 1c shows the morphology of the GQDs, which are about 5 nm in size (Figure S1, Supplementary Materials), uniformly distributed in the solution. After recombination, it can be observed from Figure 1d that the GQDs are adsorbed on the surface of the CNs, and the HRTEM image indicates that the GQDs have a lattice fringe spacing of 0.24 nm, which belongs to the (100) plane.

### 3.2. Microstructural and Optical Characterization

**Figure 2:**
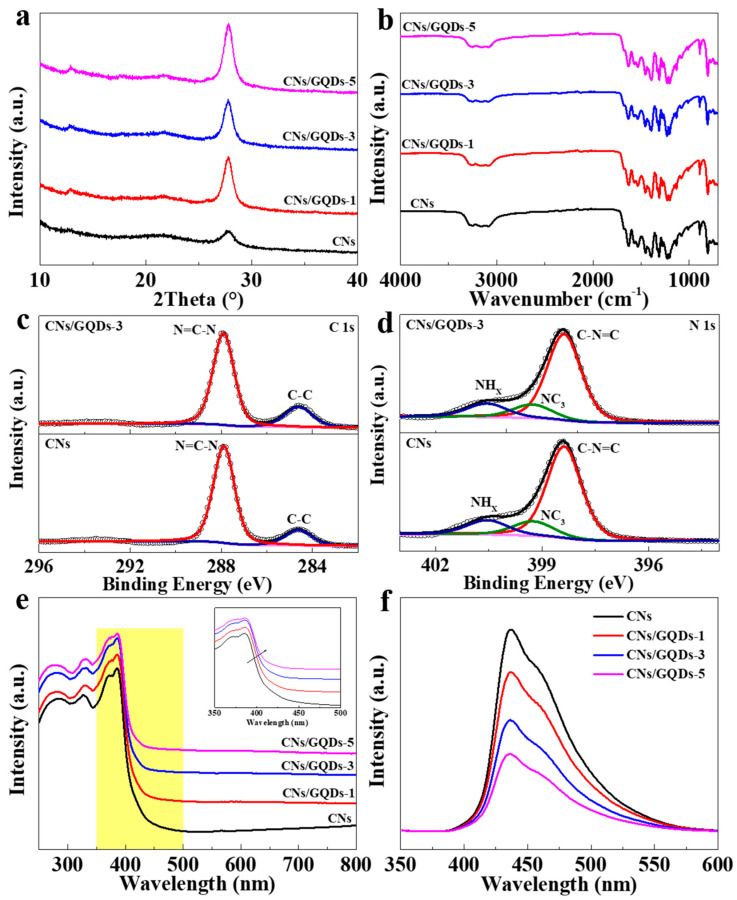
(**a**) The XRD images of CNs and CNs/GQDs; (**b**) The FTIR spectrum of CNs and CNs/GQDs; The XPS spectra of CNs and CNs/GQDs-3, (**c**) C 1 s track and (**d**) N 1s track; (**e**) The UV-vis spectrum of CNs and CNs/GQDs; and (**f**) The PL spectrum of CNs and CNs/GQDs.

The XRD spectra are used to determine the microstructural changes in the samples (Figure 2a). The CNs displays two peaks located at around 13° and 27.6° due to the (100) reflection and (002) reflection, respectively. The peak intensities of the (002) and (100) planes increased with the increase in the recombination amount of the GQDs. It was reported that the CNs are layered nanosheets with a graphite-like phase, and the layers are connected by hydrogen bonds. However, when the GQDs adsorb to the CNs surface, they will adsorb to other CNs, which leads to the superposition of layered structures, which is also the reason for the increase in the (002) intensity of the interlayer peak. Thus, the bonding between the nanosheets is tighter, due to the existence of the electrostatic adsorption force. The number of layers is also increased after annealing (Figure S2, Supplementary Materials). The FTIR was measured to study the chemical bonds’ state. Figure 2b shows a peak at 809 cm^−1^ which was caused by the out-of-plane bending vibration of the heptazine rings. The C–N heterocycles stretching results in several peaks between 900 and 1800 cm^−1^. Meanwhile, the characteristic peaks between 3000–3400 cm^−1^ can be attributed to the N–H stretching vibrations. The FTIR characteristic peaks of the CNs are similar to a previous report [24]. The CNs/GQDs samples show the same peak positions as the CNs, and there is no new peak, indicating that the CNs/GQDs have the same chemical bonds as the CNs. The XPS C 1s spectra of the CNs, which were deconvoluted into two peaks, can be attributed to the C–C and N=C–N groups located at around 284.6 and 287.88 eV, respectively (Figure 2c). The CNs/GQDs sample show no obvious binding energy shifts, implying the same chemical states of the CNs and CNs/GQDs. Due to the large number of the C–C bonds in the GQDs, the peak area ratio of the C–C group to N=C–N group increased after the formation of the CNs/GQDs. The XPS N 1s spectra of CNs are shown in Figure 2d, with three species peaks located at 398.38, 399.25, and 400.54 eV, respectively. The signal situated at 398.38 eV is ascribed to the sp^2^-hybridized nitrogen C=N-C species. Meanwhile the other two peaks at 399.25 and 400.54 eV are caused by the NC_3_ and amino groups (NH_x_), respectively. The peak positions and the peak area ratio of the CNs/GQDs samples show almost no changes at all. The results of the XPS and FTIR indicate the same chemical states exist in both the CNs and CNs/GQDs.

The optical characteristics of the CNs and CNs/GQDs are characterized with UV-vis spectrum and PL spectrum. The CNs shows an absorption edge at 419 nm (Figure 2e). As a result, the CNs have trouble absorbing visible light in the 420–800 nm range. The absorption edges of the CNs/GQDs samples are red-shifted in comparison to CNs. It can be seen from Figure S3 (Supplementary Materials) that the band gap of CNs, CNs/GQDs-1, CNs/GQDs-3, and CNs/GQDs-5 are 3.073, 3.063, 3.058, and 3.055 eV, respectively, indicating that with the increase in the quantum dot loading, the band gap of the sample is also continuously decreasing. Figure 2f shows that the CNs have the highest emission peak at 438 nm (excited at 325 nm). The emission intensity of the CNs/GQDs decreased significantly with the increased GQDs, indicating the enhanced separation efficiency of the photoinduced electron–hole pairs.

### 3.3. Surface Potential Analysis

**Figure 3:**
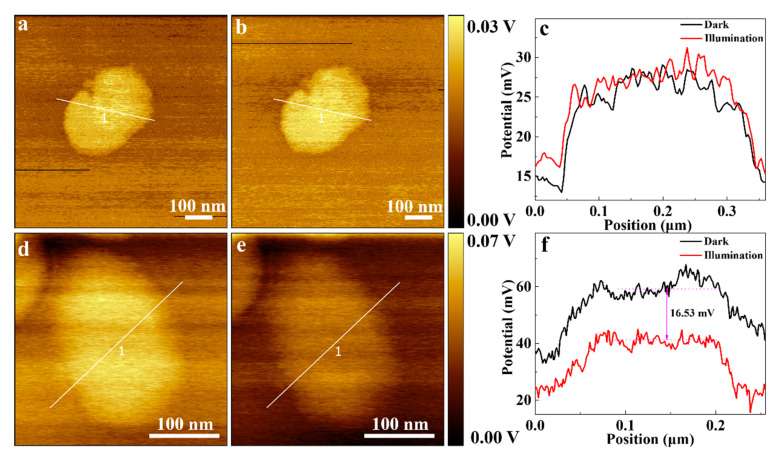
The surface potential of CNs in different conditions, (**a**) in the dark and (**b**) under illumination; (**c**) The surface potential curves along the white lines in (**a**,**b**). The surface potential of CNs/GQDs-3 (**d**) in the dark and (**e**) under illumination; (**f**) The surface potential along the white lines in (**d**,**e**).

The Scanning Kelvin Probe Microscope (SKPM) was used to measure the surface potential of the CNs and CNs/GQDs in the dark and under visible light illumination to explore the photo-enhanced catalytic process. As shown in Figure 3a–c, the CNs have a ~28 mV surface potential in dark conditions, and it shows no significant change in the visible light irradiation. The negligible surface potential change indicates that the CNs respond poorly to visible light and the recombination of the photogenerated electron–hole pairs is significant. The CNs/GQDs-3 show a 57.37 mV surface potential in dark conditions, and it decreases by 16.53 mV under visible light illumination. Previous research has shown that a more negative surface-potential signal corresponds to a higher density of electrons [44,49]. The CNs/GQDs can thus promote the photogenerated carriers’ separation under illumination, and additional photogenerated electrons can participate in the reduction process.

### 3.4. Performance Characterization

**Figure 4:**
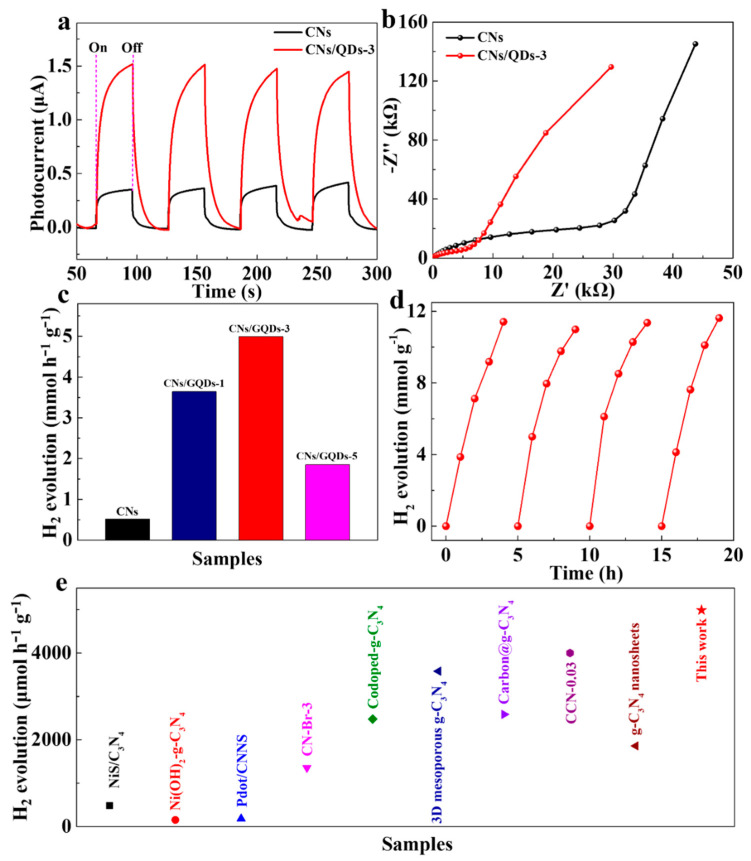
The photoelectrochemical (PEC) properties of CNs and CNs/GQDs-3. (**a**) Photocurrent and (**b**) electrochemical impedance spectroscopy (EIS) curves; (**c**) The H_2_ production rate of CNs and CNs/GQDs with Pt assistance (λ > 420 nm); (**d**) Cycling test curve of CNs/GQDs-3; (**e**) Hydrogen production capacity of CN-based catalysts [50,51,52,53,54,55,56,57,58].

The photoelectrochemical (PEC) properties were measured under visible light irradiation. As is shown in Figure 4a, the photocurrent response of the CNs/GQDs-3 is much larger than that of the CNs, and the EIS spectra in Figure 4b shows that the CNs/GQDs-3 sample shows a smaller arc radius compared with the CNs, indicating that the carriers are more easily transferred to the surface of the sample. The smaller impedance of CNs/GQDs-3 also contributes to the larger photocurrent response. To assess the photocatalytic activity of the samples, the H_2_ generation rate of CNs and CNs/GQDs were measured. Figure 4c shows that the H_2_ generation rate of CNs is 518.74 μmol g^−1^ h^−1^. The photocatalytic H_2_ evolution rate of CNs/GQDs-3 reaches 4.99 mmol g^−1^ h^−1^, which is 9.62 times greater than that of the CNs. Compared with the Pt-assisted g-C_3_N_4_-based photocatalysts that were reported so far, the CNs/GQDs samples show superior performance (Figure 4e; Table S1, Supplementary Materials). To study the wavelength dependent hydrogen generation for the CNs/GQDs-3, the hydrogen production rates at three wavelengths of 420, 550 and 600 nm were tested (Figure S4, Supplementary Materials). The AQE at 420 nm is 0.04%, which is the highest of the three wavelengths tested. To verify the stability of the photocatalyst, we tested the samples under visible light illumination for four cycles, taking a data point every hour. As shown in Figure 4d, after three cycles, the catalyst was not deactivated and still had good hydrogen evolution activity. After the test was completed, the sample was collected after the reaction and the XRD was performed, and the result shows that there was no change compared with the XRD before the reaction (Figure S5, Supplementary Materials), which indicates that the samples had good stability.

### 3.5. Mechanism Analysis

**Figure 5:**
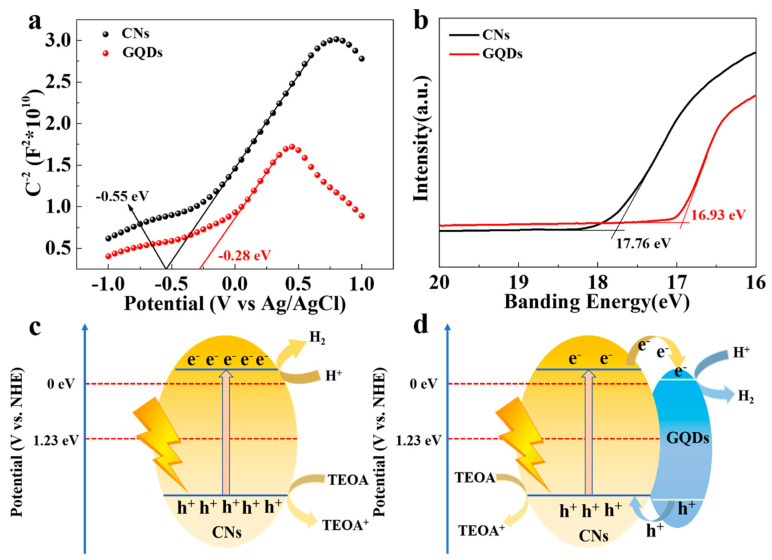
(**a**) Mott–Schottky curves of CNs and GQDs; (**b**) Ultraviolet photoelectron spectroscopy (UPS) of CNs and GQDs. The photocatalytic mechanism of (**c**) CNs and (**d**) CNs/GQDs.

We determined the conduction band positions of the CNs and GQDs, using Mott–Schottky curves. Figure 5a shows the conduction band positions of the CNs and GQDs are −0.55 and −0.28 eV, considering the Ag/AgCl electrode, respectively. The ultraviolet photoelectron spectroscopy analysis results shows that the Fermi levels of the CNs and GQDs were 3.44 and 4.27 eV below the vacuum level, respectively (Figure 5b), indicating that the CNs had a higher Fermi-level position. Therefore, the electrons flow from the CNs to the GQDs during the photocatalytic process. The band gaps of the CNs and GQDs were 3.07 and 2.80 eV, respectively, as shown in Figure S6 (Supplementary Materials). Considering the energy band position analysis, the photocatalytic reaction mechanism of the CNs and the CNs/GQDs samples was proposed. In the absence of the GQDs loading, the photoexcited electron–holes recombined rapidly, which resulted in only a small number of electrons participating in the hydrogen production reaction. After the heterojunction formation, the electrons generated by illumination in the CN sample transferred to the GQDs due to the lower conduction band position of the metallic GQDs. The TEOA selectively adsorbed the holes, thus avoiding the recombination of the photogenerated electron–hole pairs. As a result, more of the electrons could participate in the hydrogen evolution reaction, and the hydrogen production rate of CNs/GQDs was significantly improved.

## 4. Conclusions

In summary, a unique CNs/GQDs Van der Waals heterojunction was successfully prepared, which was synthesized by the electrostatic self-assembly of the atomically thin CNs and 0D GQDs. The experimental characterizations indicate that the absorption range of the sample was red-shifted when the GQDs were introduced, and the CN/GQDs sample could absorb visible light for the photocatalytic reaction. Additionally, the SPV spectroscopy confirmed that introducing the GQDs in the CNs could promote the transport of photo-induced carriers in the melon chain, hence reducing recombination in the body. This is mainly because the heterojunctions constructed with the CNs are type II heterojunctions, due to the semiconducting properties of the GODs. Thanks to the introduction of 0D GQDs, the H_2_ evolution rate of CNs was enhanced.

## Data Availability

Not applicable.

## Supplementary Materials

The following supporting information can be downloaded at: https://www.mdpi.com/article/10.3390/ma15155390/s1. Figure S1: The TEM image of GQDs; Figure S2: The AFM image of the CNs/GQDs-3, and the curve is the corresponding height profile along the white line; Figure S3. The band gap patterns of CNs; CNs/GQDs-1; CNs/GQDs-3; and CNs/GQDs-5; Figure S4: Wavelength-dependent hydrogen generation for the CNs/GQDs-3 with 10 vol% of TEOA as a sacrificial donor; Figure S5. The XRD spectra of CNs/GQDs-3 after reaction; Figure S6: Plots of the transformed Kubelka–Munk function versus the light energy of (a) CNs; and (b) GQDs; Table S1. Hydrogen production capacity of CN-based catalysts.
