# Supplementary material for: Constructing Heterogeneous Photocatalysts Based on Carbon Nitride Nanosheets and Graphene Quantum Dots for Highly Efficient Photocatalytic Hydrogen Generation
Source: Materials (Basel). 2022 Aug 5;15(15):5390. doi: 10.3390/ma15155390 (PMC9369747; doi:10.3390/ma15155390)
Supplement: Supplementary file 1 [file materials-15-05390-s001.zip › materials-1800485-supplementary.pdf]

Supplementary Materials

Article

Constructing Heterogeneous Photocatalysts Based on Carbon Nitride Nanosheets and Graphene Quantum Dots for Highly Efficient Photocatalytic Hydrogen Generation

Yong Wang [1,†], Chengxin Zeng [1,†], Yichen Liu [1], Dingyi Yang [1], Yu Zhang [2], Zewei Ren [1], Qikun Li [1], Jian Hao [3], Wen Hu [1,*], Yizhang Wu [4,*] and Rusen Yang [1,*]

1 Academy of Advanced Interdisciplinary Research, School of Advanced Materials and Nanotechnology, Xidian University, Xi'an 710126, China
2 Department of Physics, Shaanxi University of Science and Technology, Xi'an 710021, China
3 State Key Laboratory of High-Efficiency Utilization of Coal and Green Chemical Engineering, Ningxia University, Yinchuan 750021, China
4 National Laboratory of Solid State Microstructures, Collaborative Innovation Center of Advanced Microstructures and Jiangsu Provincial Key Laboratory for Nanotechnology, Nanjing University, Nanjing 210093, China
* Correspondence: huwen@xidian.edu.cn (W.H.); yzwu@smail.nju.edu.cn (Y.W.); rsyang@xidian.edu.cn (R.Y.)
† These authors contributed equally to this work.

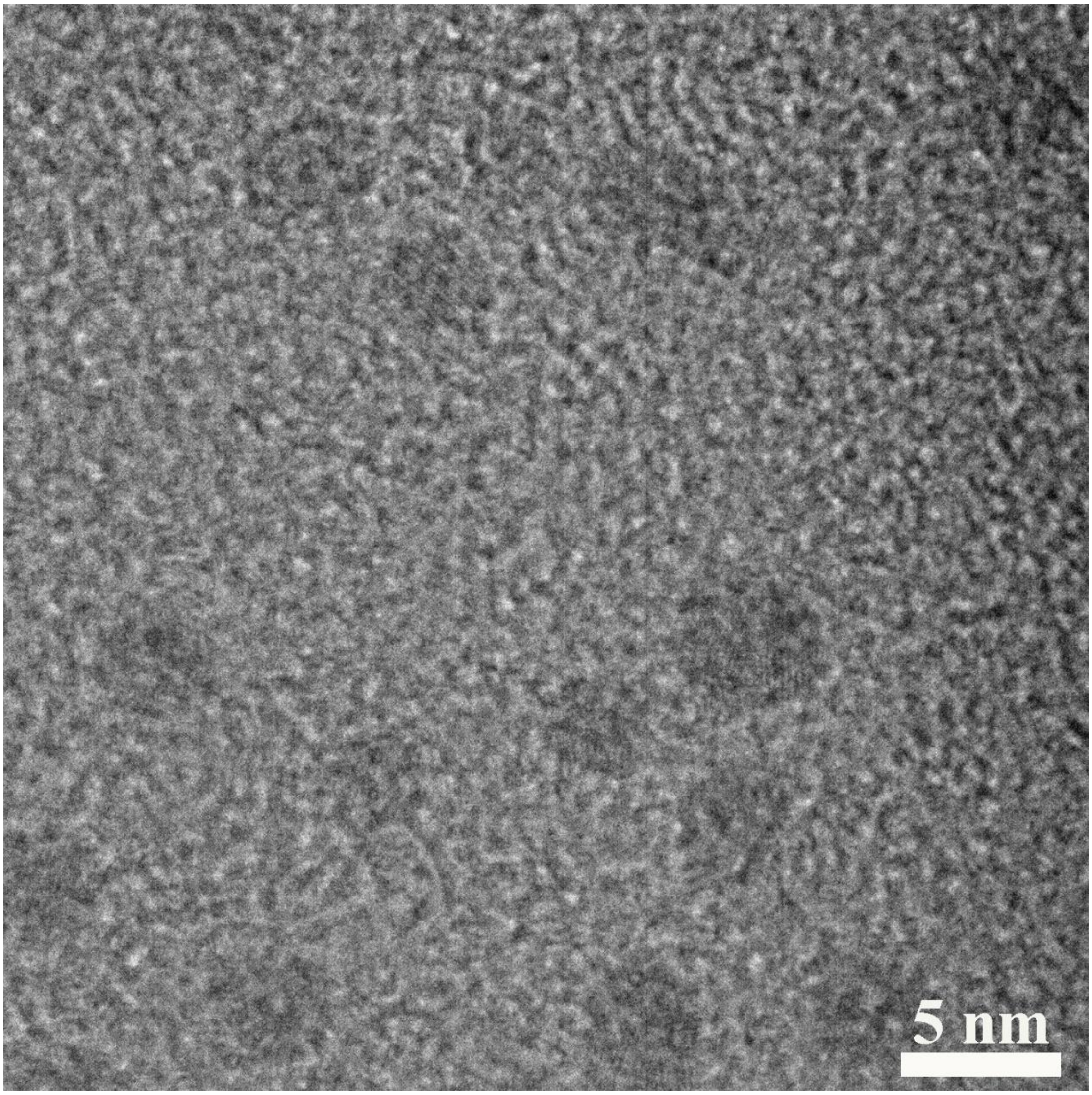

Figure S1. The TEM image of GQDs.

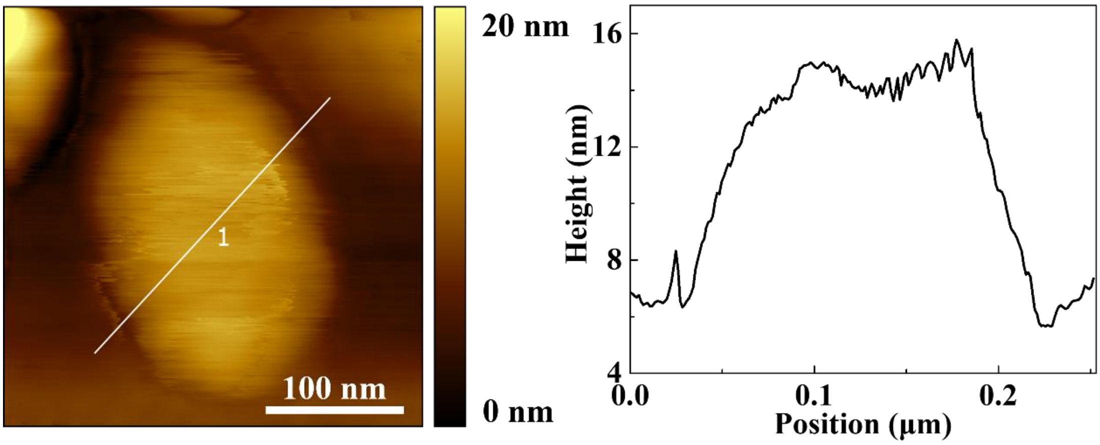

Figure S2. The AFM image of the CNs/GQDs-3, and the curve is the corresponding height profile along the white line.

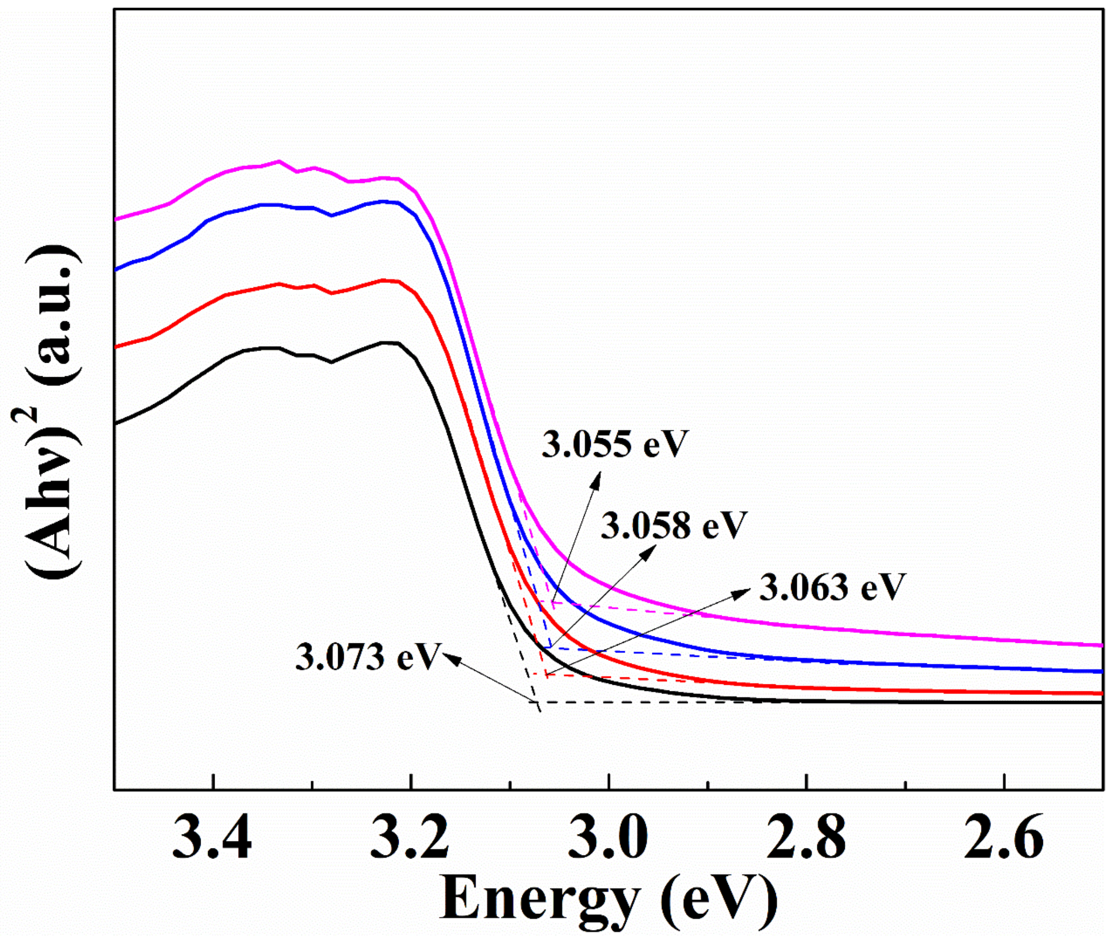

Figure S3. The band gap patterns of CNs; CNs/GQDs-1; CNs/GQDs-3; and CNs/GQDs-5.

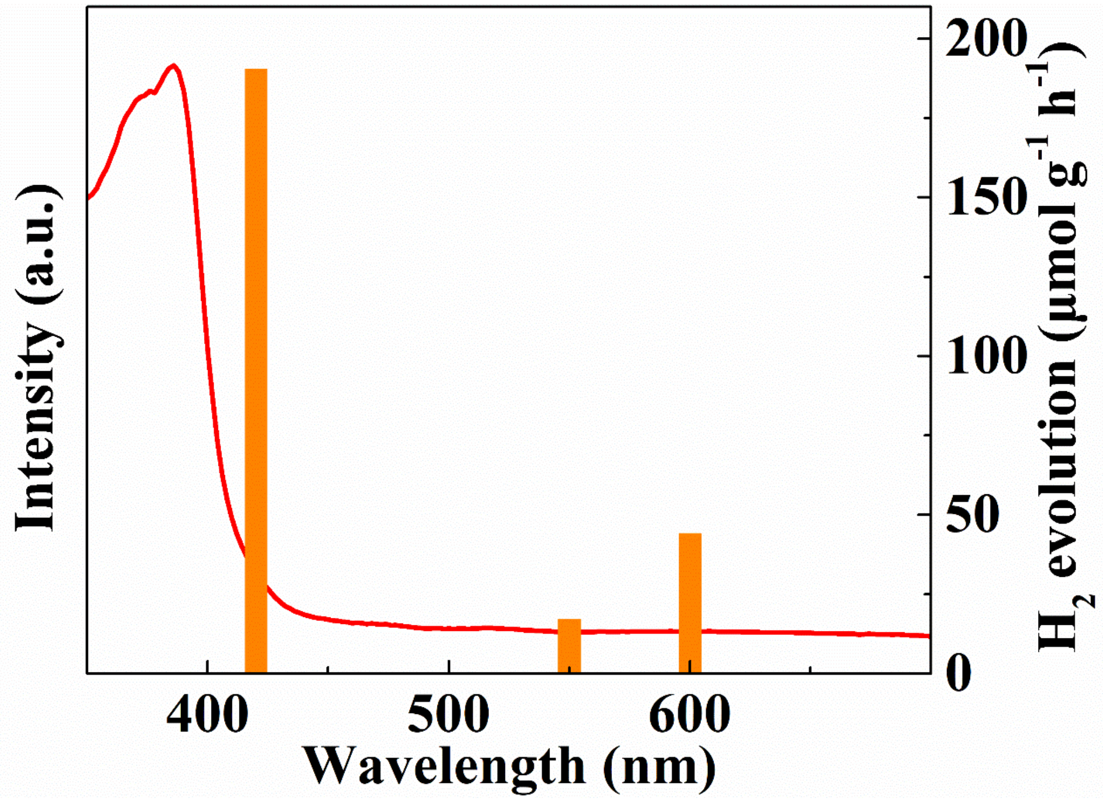

Figure S4. Wavelength-dependent hydrogen generation for the CNs/GQDs-3 with 10 vol% of TEOA as a sacrificial donor.

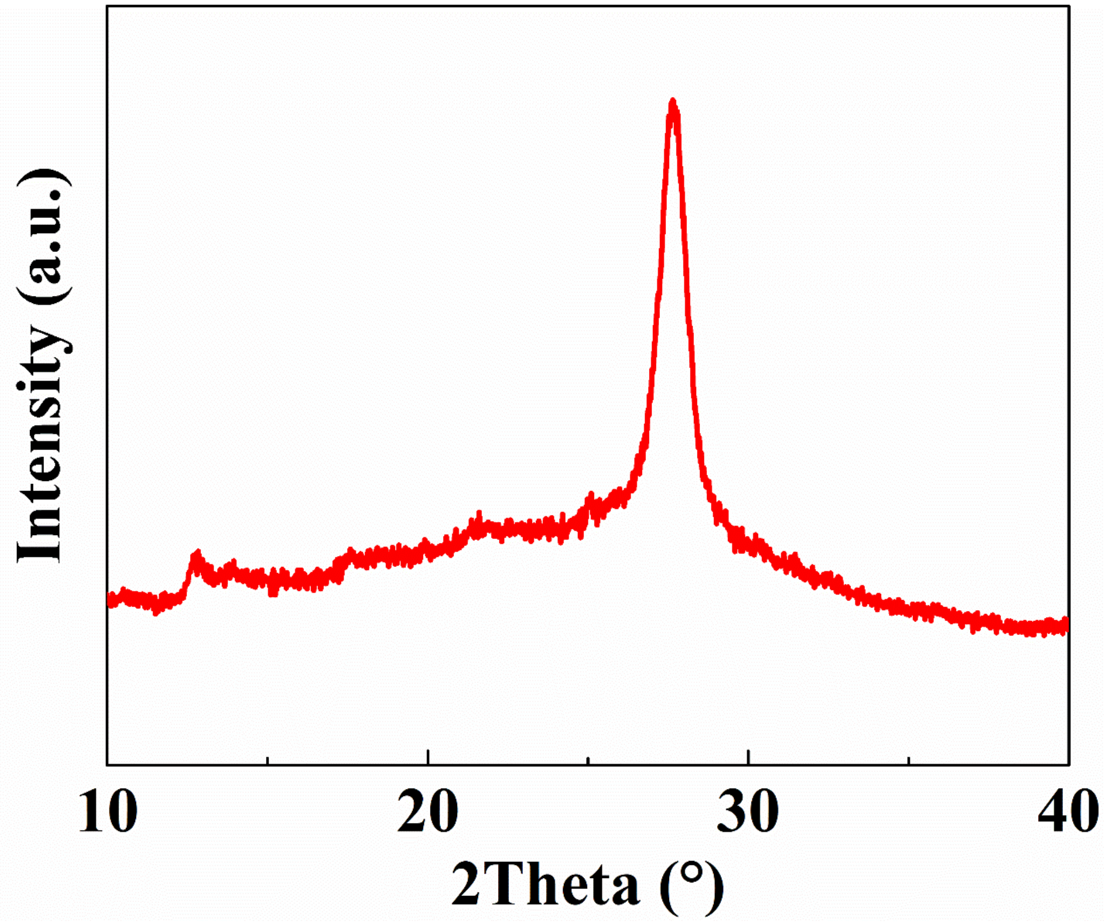

Figure S5. The XRD spectra of CNs/GQDs-3 after reaction.

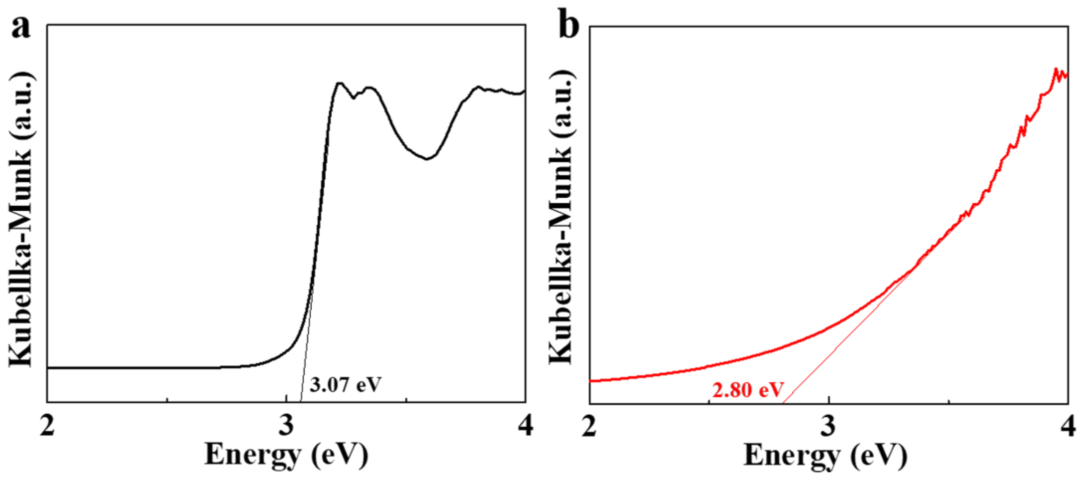

Figure S6. Plots of the transformed Kubelka–Munk function versus the light energy of (**a**) CNs; and (**b**) GQDs.

Table S1. Hydrogen production capacity of CN-based catalysts.

Reference	Sample	Light source	With or without co-catalyst	Performance
S1	NiS/C_3N_4	300 W Xenon lamp	without	482 μmol/g/h
S2	$Ni(OH)_2$-g-C_3N_4	350 W Xe arc lamp	without	152 μmol/g/h
S3	Pdot/CNNS	300 W Xe lamp	without	181 μmol/g/h
S4	CN-Br-3	300 W Xe lamp	with	1354 μmol/g/h
S5	(S, P, O)-Codoped-g-C_3N_4	300 W Xe lamp	with	2480 μmol/g/h
S6	3D mesoporous g-C_3N_4	300 W Xe lamp	with	3579 μmol/g/h
S7	Carbon@g-C_3N_4	300 W Xe lamp	with	2588.4 μmol/g/h
S8	CCN-0.03	300 W Xe lamp	with	4000 μmol/g/h

S9	g-C_3N_4 nanosheets	300 W Xe lamp	with	1860 μmol/g/h
This work	CNs/GQDs-3	300 W Xe lamp	with	4990 μmol/g/h

References

[S1] Hong J.D.; Wang Y.S.; Wang Y.B.; Zhang W.; Xu R. Noble-metal-free NiS/C_3N_4 for efficient photocatalytic hydrogen evolution from water. *ChenSusChem* **2013**, 6, 2263-2268.

[S2] Yu J.G.; Wang S.H.; Cheng B.; Lin Z.; Huang F. Noble Metal-free $Ni(OH)_2$/g-C_3N_4 composite photocatalyst with enhanced visible-light photocatalytic H_2-production activity. *Catal. Sci. Technol.* **2013**, 3, 1782-1789.

[S3] Zhou W.; Jia T.; Zhang D.Q.; Zheng Z.K.; Hong W.; Chen X.D. The enhanced co-catalyst free photocatalytic hydrogen evolution and stability based on indenofluorene-containing donor-acceptor conjugated polymer dots/g-C_3N_4 nanosheets heterojunction. *Appl. Catal. B：Environ.* **2013**, 259, 118067.

[S4] Liu C.Y.; Huang H.W.; Ye L.Q.; Yu S.X.; Tian N.; Du X.; Zhang T.R.; Zhang Y.H. Intermediate-mediated strategy to horn-like hollow mesoporous ultrathin g-C_3N_4 tube with spatial anisotropic charge separation for superior photocatalytic H_2 evolution. *Nano Energy* **2017**, 41, 738–748.

[S5] Liu Q.Q.; Shen J.Y.; Yu X.H.; Yang X.F.; Liu W.; Yang J.; Tang H.; Xu H.; Li

H.M.; Li Y.Y.; Xu J.S. Unveiling the origin of boosted photocatalytic hydrogen evolution in simultaneously (S, P, O)-codoped and exfoliated ultrathin g-C_3N_4 nanosheets. *Appl. Catal. B：Environ.* **2017**, 248, 84-94.

[S6] Tian N.; Zhang Y.H.; Li X.W.; Xiao K.; Du X.; Dong F.; Waterhouse G.; Zhang T.R.; Huang H.W. Precursor-reforming protocol to 3D mesoporous g-C_3N_4 established by ultrathin self-doped nanosheets for superior hydrogen evolution. *Nano Energy* **2017**, 38, 72–81.

[S7] Ma L.T.; Fan H.Q.; Fu K.; Lei S.H.; Hu Q.Z.; Huang H.T.; He G.P. Protonation of graphitic carbon nitride (g-C_3N_4) for an electrostatically self-assembling carbon@g-C_3N_4 core−shell nanostructure toward high hydrogen evolution. *ACS Sustainable Chem. Eng.* **2017**, 5, 7093−7103.

[S8] Yuan J.L.; Liu X.; Tang Y.H.; Zeng Y.X.; Wang L.L.; Zhang S.Q.; Cai T.; Liu Y.T.; Luo S.L.; Pei Y.; Liu C.B. Positioning cyanamide defects in g-C_3N_4: engineering energy levels and active sites for superior photocatalytic hydrogen evolution. *Appl. Catal. B：Environ.* **2018**, 237, 24-31.

[S9] Yang S.B.; Gong Y.J.; Zhang J.S.; Zhan L.; Ma L.L.; Fang Z.Y.; Vajtai R.; Wang X.C.; Ajayan P. Exfoliated graphitic carbon nitride nanosheets as efficient catalysts for hydrogen evolution under visible light. *Adv. Mater.* **2013**, 25, 2452–2456.
